# Use of Non-Contrast-Enhanced MR Angiography to Assess Recanalization after Uterine Artery Embolization

**DOI:** 10.3390/jcm12052053

**Published:** 2023-03-05

**Authors:** Juliana Yumi Ishisaki, Hitomi Kato, Yoshiki Kuwatsuru, Hiroshi Toei, Ayako Hoshina, Naoki Takemasa, Masafumi Arai, Ryohei Kuwatsuru

**Affiliations:** 1 Department of Radiology, Graduate School of Medicine, Juntendo University, 2-1-1 Hongo, Bunkyo City, Tokyo 113-8421, Japan; 2 Department of Radiology, School of Medicine, Juntendo University, 2-1-1 Hongo, Bunkyo City, Tokyo 113-8421, Japan

**Keywords:** uterine artery, uterine artery embolization, magnetic resonance angiography

## Abstract

The purpose of this study was to examine the use of non-contrast-enhanced MR angiography (MRA) for assessing recanalization of uterine arteries (UAs) after uterine artery embolization (UAE) for symptomatic fibroids. Pre-procedural and follow-up unenhanced MRA images of 30 patients were reviewed, and the extent to which the UAs could be visualized was classified on a 4-point scale. An increase in the score between consecutive time points indicates that a previously inconspicuous segment of the UA became visible on follow-up images. Patients were divided into two groups according to the presence (or absence) of recanalization. The median UA visualization score at each follow-up was significantly lower than that at baseline (*p* < 0.01), but there was no significant difference between the scores of the follow-up images. Recanalization was detected in 63% (19/30) of patients. In these patients, the mean decrease in uterine and largest fibroid volume at 12 months after UAE was inferior to the mean decrease in patients for whom recanalization was not detected. Based on MRA assessment, recanalization after UAE occurred in 63% of patients but did not compromise the reduction in uterine and dominant fibroid volumes within 12 months after UAE.

## 1. Introduction

Magnetic resonance imaging (MRI) plays an important role in pre-procedural and follow-up examinations of patients with symptomatic uterine fibroids who are treated with uterine artery embolization (UAE) [1,2,3,4,5]. Before UAE, this is the most accurate imaging method to assess the number of fibroids, their size and location. After the procedure, MRI can be used to evaluate the technical success of embolization, changes in volume of uterus and tumor, and to image potential complications, such as transcervical fibroid expulsion [1,2,3,4,5].

Technical advances in imaging acquisition now permit excellent visualization of the pelvic vasculature, even without the use of contrast medium [6,7,8,9,10]. Non-contrast-enhanced (NCE) magnetic resonance angiography (MRA) imaging before UAE is useful to demonstrate the origin of uterine arteries (UAs) from type I to type IV and its three-dimensional configuration, and to depict possible extra-collateral feeders to the tumor, such as the ovarian artery [7,8,10]. However, few studies have used NCE-MRA to examine changes in UA recanalization after UAE [11]. Here, we used NCE-MRA obtained at four time points to investigate luminal recanalization of UAs after UAE. From the aspect of patient care, recanalization affects two important points related to UAE: First, recanalization is necessary if embolization needs to be repeated in the case of inadequate symptom control, and second, recanalization is needed for maintenance of endometrial and myometrial perfusion after UAE [12,13].

In this study, we retrospectively reviewed pre- and post-procedural non-contrast-enhanced MRA images from patients who underwent UAE for symptomatic uterine fibroids at our institution. We focused on the assessment of UA recanalization and its influence on UAE outcomes.

## 2. Materials and Methods

### 2.1. Subjects

This research was approved by the institutional review board, and because of the retrospective nature of this study, the requirement for informed consent was waived. From December 2018 through July 2020, fifty-two consecutive patients underwent UAE in our department for the treatment of symptomatic uterine fibroids. As part of our protocol, all patients scheduled for UAE undergo pre-procedural MR imaging examination, including NCE-MRA, and are asked to return for follow-up imaging after 3, 6, and 12 months. In this study, we included only patients who underwent all MRI/MRA examinations with a 3-T MR unit (Vantage Galan 3T/ZGO; Canon Medical Systems, Otawara, Japan).

**Figure 1:**
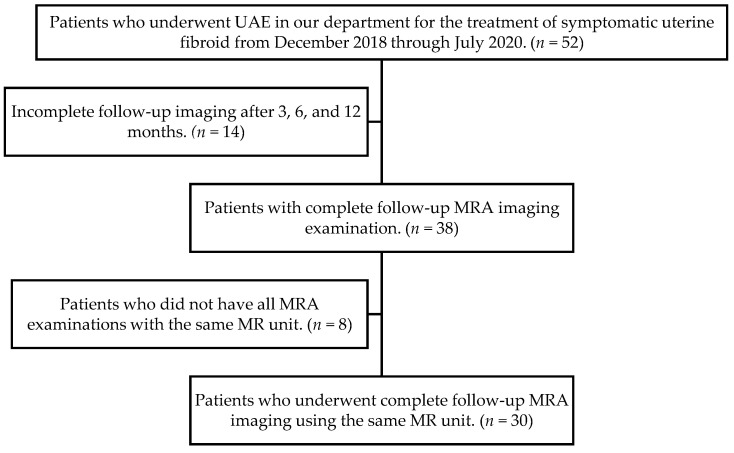
Flowchart of patient selection.

Ultimately, 30 patients (mean age, 46 years; range, 42–49 years) met the inclusion requirements and constituted our study group (Figure 1). Main reported symptoms were heavy menstrual bleeding, dysmenorrhea, abdominopelvic pain or pressure, and polyuria. Symptoms were evaluated by a questionnaire administered before and four times after the treatment (1, 4, 7, and 12 months). Hypermenorrhea and dysmenorrhea were each evaluated on a scale of 0 to 10. Each patient’s satisfaction with treatment was assessed by using a 5-point scale (1, very unsatisfied; 2, unsatisfied; 3, neither satisfied nor unsatisfied; 4, satisfied; 5, very satisfied). Patients were also asked to rate the likelihood of recommending the procedure to someone, on a 4-point scale (1, would not recommend; 2, not sure; 3, probably would recommend; 4, definitely would recommend).

### 2.2. Imaging Protocol

All examinations were conducted in a 3-T MR unit, using a pair of phased-array coils (16 channels with 16 elements: Atlas Speeder Body Coil combined with Atlas Speeder Spine Coil; Canon Medical Systems, Otawara, Japan) placed at the front and back of the abdomen. Our imaging protocol for patients undergoing UAE includes sagittal and axial fast-spin echo T2-weighted imaging (T2WI), sagittal T1-weighted imaging (T1WI), sagittal T1WI with fat saturation, diffusion-weighted imaging (b-value = 1000), apparent diffusion coefficient mapping, and NCE-MRA. The true steady-state free precession combined with time–spatial labeling inversion pulse with peripheral-pulse gating was used to obtain unenhanced three-dimensional reconstructions of the uterine and tumor vasculature. A detailed description of the technique used to obtain NCE-MRA images in our service has been described elsewhere [10].

### 2.3. UAE Technique

All procedures were performed by the same principal interventional radiologist (R.K., who has 19 years of experience in performing UAE). A 4-French (Fr) vascular sheath (Medikit Supersheath; Medikit Co., Ltd., Tokyo, Japan) was placed into the right common femoral artery under local anesthesia. The tip of a PIG-S4 catheter (Medikit Angiography Catheter MH; Medikit Co., Ltd., Tokyo, Japan) was placed in the abdominal aorta (L2 level) by using an angled guide wire (Radiofocus Guide Wire M; Terumo Co., Ltd., Tokyo, Japan), and initial aortography using iopromide 300 (300 mg of iodine per milliliter, Ultravist^®^, Bayer AG, Leverkusen, Germany) was performed by using a digital subtraction angiography unit (INFX-8000C/JL; Canon Medical Systems, Tokyo, Japan) to assess the bifurcation of the internal iliac artery, identify both UAs, check the tumor staining, and detect possible additional tumor feeder vessels, such as the ovarian artery. The amount of contrast medium used in arteriography performed before and after UAE was 24 mL each and the injection rate was 8 mL/second. Then, the catheter was exchanged for a 4-Fr MOHRI-1, catheter (Medikit Angiography Catheter; Medikit Co., Ltd. Tokyo, Japan), which was advanced through the left internal iliac branch and its anterior division until the beginning of the UA. From this point, superselective catheterization to the proximal ascending segment of the UA was performed by using a microcatheter (2.6-Fr Masters HF; ASAHI Intecc, Aichi, Japan) over a RUN&RUN micro-guidewire (0.016 inch; Piolax Medical Devices, Kanagawa, Japan). The territory supplied by the target vessel was confirmed, and embolic material was administered to the site.

Embolization was achieved by deploying tris-acryl gelatin microspheres (Embosphere; Merit Medical, UT, USA) that were 500–700 μm and/or 700–900 μm in diameter. The microspheres are provided in a prefilled syringe containing 2 mL of embolic particles and 9 mL of sterile physiological saline. To deliver the contents, 9 mL of contrast was added to the syringe and mixed thoroughly to suspend the microspheres in the solution. Embolization was terminated once the proximal ascending segment of the UA was no longer visible on the selective arteriogram during the interval of 3–5 heartbeats, after the injection of 2 mL of contrast medium. Thereafter, the same sequence was performed on the right UA through a unilateral approach. Finally, aortography was performed, and once the absence of a peritumoral arterial plexus and slow antegrade flow in the main UAs were confirmed, the procedure was considered finished. All procedures were performed successfully. In one patient, the pre-procedural MRA, abdominal aortogram, and selective arteriogram revealed no significant contribution from the right UA to the tumor supply; therefore, unilateral embolization was performed on the left UA only.

### 2.4. Visual Classification of Uterine Arteries

**Figure 2:**
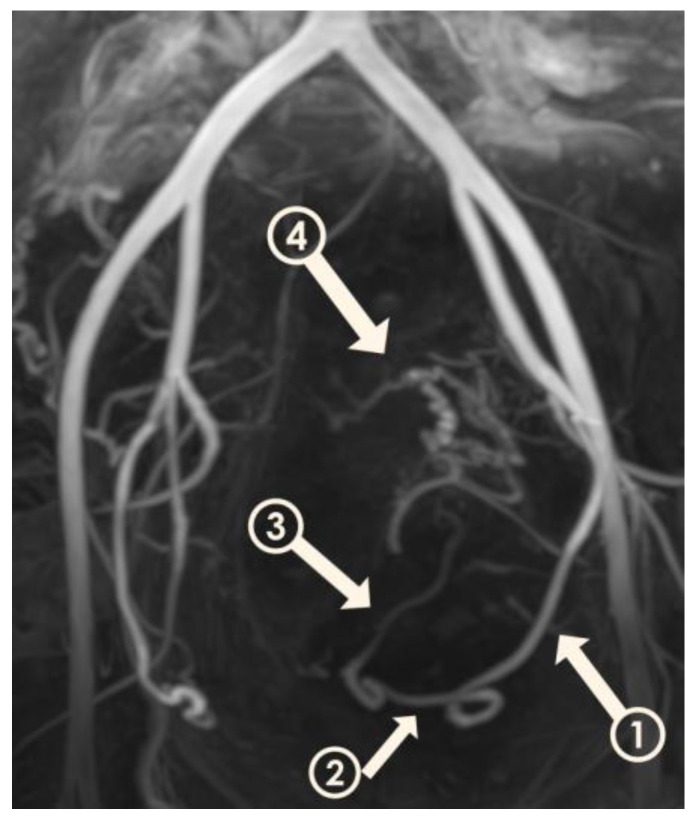
Three-dimensional non-contrast-enhanced magnetic resonance angiography image from a 49-year-old woman. The model is rotated to show the left uterine artery and its segments. Descending segment (arrow 1); transverse segment (arrow 2); ascending segment (arrow 3); peritumoral plexus (arrow 4).

**Figure 3:**
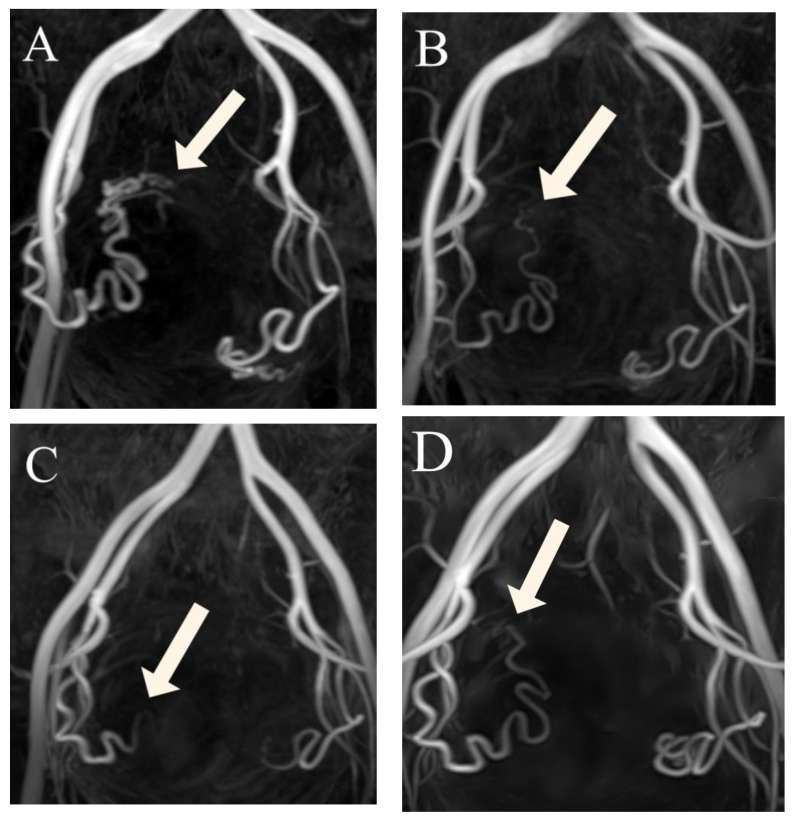
(**A**–**D**) Three-dimensional non-contrast-enhanced magnetic resonance angiography image from a 49-year-old woman, suggesting luminal recanalization of the uterine arteries after uterine artery embolization. (**A**) Before uterine artery embolization: visualization of the right UA (arrow) forming the peritumoral plexus (score 4). (**B**) Three months after uterine artery embolization: visualization of the right uterine artery (arrow) until the ascending segment (score 3). (**C**) Six months after uterine artery embolization: visualization of right uterine artery (arrow) until the transverse segment (score 2). (**D**) Twelve months after uterine artery embolization: visualization of the right uterine artery (arrow) until the ascending segment (score 3).

**Figure 4:**
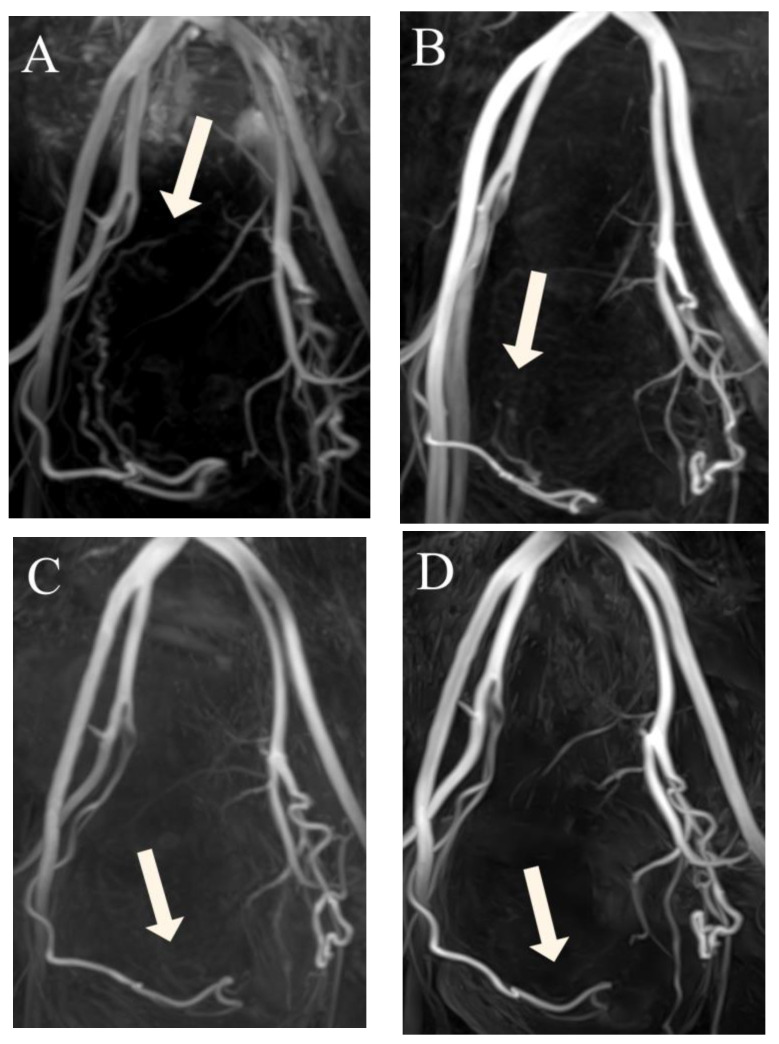
(**A**–**D**) Three-dimensional non-contrast-enhanced magnetic resonance angiography image from a 48-year-old woman, suggesting that luminal recanalization of the uterine arteries did not occur after uterine artery embolization. (**A**) Before uterine artery embolization: visualization of the right UA (arrow) forming the peritumoral plexus (score 4). (**B**) Three months after uterine artery embolization: visualization of the right uterine artery (arrow) until the ascending segment (score 3). (**C**) Six months after uterine artery embolization: visualization of right uterine artery (arrow) until the transverse segment (score 2). (**D**) Twelve months after uterine artery embolization: visualization of the right uterine artery (arrow) until the transverse segment (score 2).

Visualization of UAs was scored by two radiologists in consensus at four time points: before UAE, at 3 months after UAE, at 6 months after UAE, and at 12 months after UAE. A 4-point scale (Figure 2) was established for classifying the degree of visualization of the right and left UAs individually: 1, visualization until the descending segment; 2, visualization until the transverse segment; 3, visualization until the ascending segment; and 4, visualization until the peritumoral plexus. By assessing the variation in scores at these four time points, we were able to estimate the occurrence of recanalization in our patients (Figure 3A–D). We used this information to divide patients into two groups: those in whom scores for both right and left UAs remained the same or decreased over time (Group 1) (Figure 4A–D), and patients whose scores increased (Group 2), suggesting luminal recanalization. If either of the UAs had an increase in score, the patient was classified as Group 2.

### 2.5. Volume Measurement

**Figure 5:**
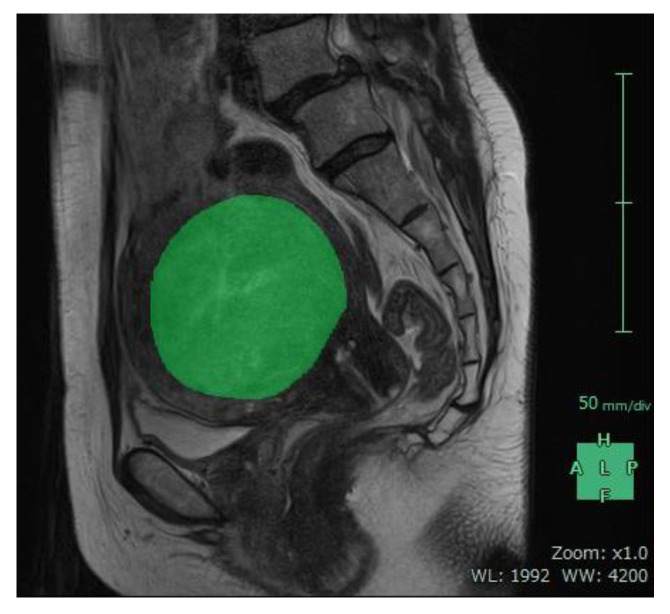
Sagittal MR image from a 48-year-old woman obtained before uterine artery embolization. The margins of the uterine tumor were delineated in sequential sagittal slices similar to those shown in green (mask).

**Figure 6:**
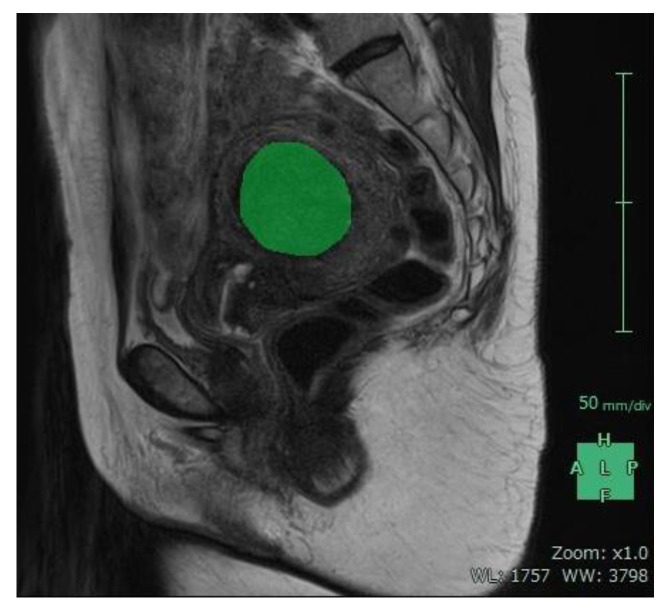
Sagittal image of the same patient 12 months after uterine artery embolization. The margins of the uterine tumor were delineated in sequential sagittal images similar to those shown in green (mask).

**Figure 7:**
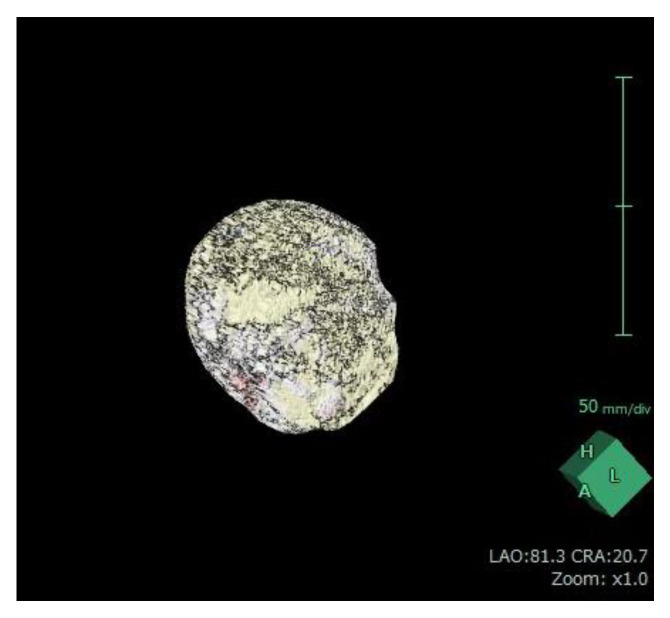
Three-dimensional reconstruction of the uterine fibroid before uterine artery embolization obtained using Synapse Vincent software.

**Figure 8:**
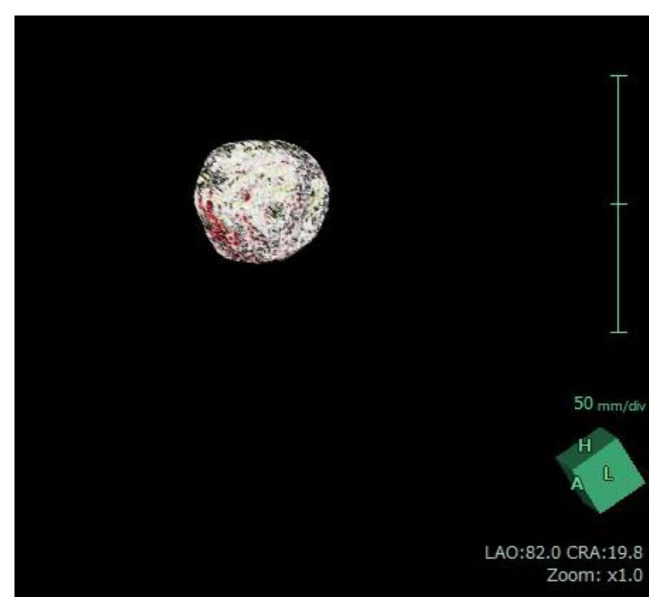
Three-dimensional reconstruction of the uterine fibroid 12 months after uterine artery embolization obtained using Synapse Vincent software.

For each patient, baseline (Figure 5) and 12-month post-procedural (Figure 6) sagittal T2-weighted images were imported into 3D volume analyzer software (SYNAPSE; Fujifilm Corporation, Tokyo, Japan). After a radiologist had delineated the margins of the uterus and the largest tumor in sequential sagittal slices, the program automatically calculated their total volumes (Figure 7 and Figure 8). The same investigator (J.Y.I.) performed all measurements, and one other investigator (R.K.) reviewed them all.

Uterine fibroid volume measurement performed with Synapse Vincent software (Fujifilm Corporation, Tokyo, Japan).

Figure 7 and Figure 8 were obtained from the same patient of Figure 5 and Figure 6. 

### 2.6. Statistical Analysis

Statistical analysis was performed by using commercially available software (SPSS Statistics for Windows, version 26.0, IBM, NY, USA). Normally distributed data are reported as the mean value ± 1 standard deviation, and non-normally distributed data are given as median and interquartile range (IQR). Normality was checked using the Shapiro–Wilk test. Friedman’s two-way analysis test for related samples was used to compare the distribution of scores from the MRAs at baseline, 3 months, 6 months, and 12 months. Variation between the two groups of patients in volume of uterus and largest fibroid was compared by using the Mann–Whitney U test. The threshold for significance was set to *p* < 0.05.

## 3. Results

Embolization was successfully performed in all cases. Comparison of baseline characteristics of the groups showed no significant difference in age (*p* = 0.64), initial volume of largest tumor (*p* = 0.70), or initial uterine volume (*p* = 0.58). Chronological analysis of MRA images revealed 11 cases with bilateral absence of recanalization (Group 1), and 19 cases of luminal recanalization (Group 2). Among the cases in Group 2, bilateral recanalization of UAs was found in 9 patients. In Group 1, 91% (10/11) of patients showed improvement of symptoms after treatment. In Group 2, 84% (16/19) of patients showed improvement of symptoms. In Group 2, two patients underwent additional treatment (total laparoscopic hysterectomy). Comparison of results in the two groups is shown in Table 1.

Among the 59 UAs analyzed in this study, MRA at baseline allowed visualization until the peritumoral plexus in 40 UAs, whereas similarly complete visualization was seen for only eight UAs when MRA was performed at 3 and 6 months after UAE, and for nine UAs when performed at 12 months. The numbers of UA segments visualized at each time point are summarized in Table 2.

The median (IQR) of the scores for visualization of UAs was 4 (IQR: 3–4) in baseline MRA images, and 3 (IQR: 2–3) in 3-, 6-, and 12-month MRA images. The extent of visualization of the UAs decreased significantly between baseline and follow-up images (*p* < 0.01), but there was no difference in extent of visualization between the follow-up images: between 3- and 6-month images, *p* = 0.83; 3- and 12-month images, *p* = 0.91; and 6- and 12-month images, *p* = 0.91.

Median uterine volume among all patients was 742 cm^3^ (IQR: 574–1169 cm^3^) before UAE and 519 cm^3^ (range, 315–798 cm^3^) at 12 months after UAE, corresponding to a mean volume reduction of 37% during this period. Comparison of the two patient groups showed that uterine volume was decreased by a mean of 39% in Group 1 but by 36% in Group 2. However, these values did not differ significantly between the two groups (*p* = 1.00).

Median volume of the dominant fibroid was 326 cm^3^ (IQR: 197–551 cm^3^) before UAE but 189 cm^3^ (IQR: 79–359 cm^3^) at 12 months after UAE, representing a mean decrease in volume of 45% during this period. The mean reduction in dominant fibroid volume was 49% for Group 1, compared with 42% in patients with recanalization. Again, these values did not differ significantly between groups (*p* = 0.44).

In one case, we performed unilateral embolization of the left UA with good results. The patient reported improvement of symptoms 1 month after the treatment, and had a reduction of 45% in uterine volume, and 64% for the largest tumor within 1 year.

## 4. Discussion

The present study demonstrated that, based on MRA findings, recanalization of UAs within 12 months of UAE using tris-acryl gelatin microspheres occurred frequently but did not seem to compromise the efficacy of UAE for the treatment of uterine fibroids. Our results are consistent with those of prior studies that used contrast-enhanced MRI to assess recanalization of UAs after UAE and its influence on overall outcomes [14,15].

Katsumori et al. [14] used contrast-enhanced MRA images obtained at baseline and after 4 months to study recanalization after UAE using gelatin sponge particles. Although recanalization occurred in the majority (88%) of embolized UAs, contrast-enhanced MRI performed 1 week after UAE showed that the infarction rate exceeded 90% in the largest tumors in all patients. Furthermore, 94% of patients demonstrated clinical improvement at 4 months after UAE. In their study, one limitation that the authors pointed out was the difficulty in assessing recanalization in arteries distributed beyond the ascending segment of the UA using 1.5-T contrast-enhanced MRA imaging [14]. In our study, the superiority of 3-T NCE-MRA to show the arteries beyond the ascending segment (forming the peritumoral plexus which feeds the uterine fibroid) in 68% of the arteries evaluated (40 of 59 UAs) was demonstrated. This comparison shows the importance of the field strength of the MR machine and the imaging technique to vascular studies and supports our decision to include only patients evaluated with a 3-T MR unit in this investigation. A similar study conducted by Das et al. [15] involved 50 patients who underwent UAE using non-spherical polyvinyl alcohol particles. At 6 months after the procedure, MRA detected recanalization in 90% of cases. However, recanalization had no significant effect on patient outcome in terms of fibroid infarction rates, volume reduction, or symptoms after UAE [15].

For studies that included contrast-enhanced MRI data [14,15], UA recanalization may suggest not only the recanalization of fibroid-feeding arteries but also reperfusion of normal uterine parenchyma. In our department, we do not perform contrast-enhanced imaging as routine for all patients, so we could not further assess uterine reperfusion after UAE. This is a limitation of our study. In our experience, assessment of the peritumoral plexus on NCE-MRA images allows us to estimate the infarction of fibroids after UAE: Disappearance of the peritumoral plexus in post-procedural NCE-MRA images suggests successful infarction of fibroids, while a remaining peritumoral plexus suggests otherwise. We acknowledge that this method is not as accurate as using contrast-enhanced MRA to evaluate tumor and uterine perfusion after UAE, but we do find a higher reduction in uterine and fibroid volume for the group of patients in which the peritumoral plexus is not visible on post-procedural MRA. We plan to address this topic in a future study.

Despite the well-known safety of UAE, its effects and timing need further clarification. Whether the target vessels will recanalize (or not) after UAE is unpredictable, and when recanalization might begin after UAE cannot be estimated [16]. In this regard, we want to highlight the importance of performing a detailed chronologic assessment to assess vascular recanalization. Without the 3- and 6-month MRA images, 52% of the recanalization cases would have gone unnoticed in our investigation. In such cases, although the score at 12 months was equal to, or lower than, that at baseline (giving a false perception that recanalization did not happen), assessment of the 3- and 6-month MRA images revealed that within the 1-year interval, there was a decrease and subsequent increase in the scores in some patients, suggesting that recanalization had in fact occurred. Therefore, we believe that the assessment at four time points is a strong feature of our study. We understand that this pattern is insufficient for predicting or generalizing the timing of UA recanalization after UAE, but it does demonstrate that we must be aware of earlier events when assessing chronological follow-up images to study embolization-induced vascular changes.

We acknowledge several limitations to our current study. First, it was a retrospective analysis of a small number of patients. Selecting only patients for whom all four exams were performed with the same MR unit (3-T) unavoidably reduced our sample size. Furthermore, we acknowledge that the lack of contrast administration results in limited information about tumor infarction and uterine perfusion after UAE. Non-contrast-enhanced MRA may struggle in areas where there is perfusion but slow-flow. It would therefore be useful to compare unenhanced MRA findings with contrast-enhanced MRA or angiography in future studies. Significant improvement of imaging methods and safety concerns associated with gadolinium-based contrast agents (such as deposition in the brain and nephrogenic systemic fibrosis in patients with chronic renal disease) have been the main drivers of our study [9,10,17,18]. We expect that our findings will motivate other researchers to expand the literature focusing on non-contrast-enhanced imaging before and after embolization.

## 5. Conclusions

In conclusion, our study demonstrated that visualization of UA segments (mainly the peritumoral plexus) on MRA images significantly decreased after UAE. Non-contrast-enhanced MRA allowed us to visually classify all UAs in this study and estimate the occurrence of luminal recanalization after UAE among our cases. However, further studies are necessary to confirm our findings.

## Figures and Tables

**Table 1:** Comparison between Group 1 and Group 2.

	Group 1	Group 2	*p* Values
Number of cases	11	19	
Percentage reduction in uterus volume	39% (±11.6) ^a^	36% (±14.4) ^a^	1.00
Percentage reduction in volume of largest tumor	49% (±18.4) ^a^	42% (±20.8) ^a^	0.44
Symptom improvement (number of cases)	91% (10/11)	84% (16/19)	0.76
Hypermenorrhea before UAE (scale 0–10)	10 (IQR: 10–10) ^b^	10 (IQR: 8–10) ^b^	0.25
Hypermenorrhea after UAE (scale 0–10)	5.7 (±2.4) ^a^	5.7 (±1.7) ^a^	0.96
Dysmenorrhea before UAE (scale 0–10)	7 (IQR: 5.5–10) ^b^	5 (IQR: 3–7) ^b^	0.06
Dysmenorrhea after UAE (scale 0–10)	3.9 (±2.4) ^a^	3.5 (±2.3) ^a^	0.83
Satisfaction with treatment (scale 1–5)	4 (IQR: 4–5) ^b^	4 (4–4.5) ^b^	0.55
Likelihood of recommending UAE (scale 1–4)	3 (IQR: 3–3) ^b^	3 (IQR: 2.5–3) ^b^	0.47

UAE, Uterine artery embolization; IQR, interquartile range. ^a^ Mean value (± standard deviation); ^b^ Median (interquartile range). Note: In the table, Group 1 is group with no imaging findings of recanalization. Group 2 is group with findings of recanalization; 9 of 19 had bilateral recanalization. The percentage reduction in volume correspond to 12-month measurement compared to baseline measurement.

**Table 2:** Visualization of uterine artery segments before and after uterine artery embolization.

UA Segment	Before UAE	3 Months after UAE	6 Months after UAE	12 Months after UAE
Peritumoral plexus	40	8	8	9
Ascending segment	12	28	26	26
Transverse segment	6	18	20	18
Descending segment	1	5	5	6
Total	59	59	59	59

UAE—uterine artery embolization; UA—uterine artery.

## Data Availability

The data that support the findings of this study are available from the corresponding author (R.K.) upon reasonable request.
